# Compartmentalized Human Immunodeficiency Virus Type 1 Originates from Long-Lived Cells in Some Subjects with HIV-1–Associated Dementia

**DOI:** 10.1371/journal.ppat.1000395

**Published:** 2009-04-24

**Authors:** Gretja Schnell, Serena Spudich, Patrick Harrington, Richard W. Price, Ronald Swanstrom

**Affiliations:** 1 Department of Microbiology and Immunology, University of North Carolina at Chapel Hill, School of Medicine, Chapel Hill, North Carolina, United States of America; 2 Department of Neurology, University of California at San Francisco, San Francisco General Hospital, San Francisco, California, United States of America; 3 Lineberger Comprehensive Cancer Center, University of North Carolina at Chapel Hill, School of Medicine, Chapel Hill, North Carolina, United States of America; 4 UNC Center for AIDS Research, University of North Carolina at Chapel Hill, School of Medicine, Chapel Hill, North Carolina, United States of America; NIH/NIAID, United States of America

## Abstract

Human immunodeficiency virus type 1 (HIV-1) invades the central nervous system (CNS) shortly after systemic infection and can result in the subsequent development of HIV-1–associated dementia (HAD) in a subset of infected individuals. Genetically compartmentalized virus in the CNS is associated with HAD, suggesting autonomous viral replication as a factor in the disease process. We examined the source of compartmentalized HIV-1 in the CNS of subjects with HIV-1–associated neurological disease and in asymptomatic subjects who were initiating antiretroviral therapy. The heteroduplex tracking assay (HTA), targeting the variable regions of *env*, was used to determine which HIV-1 genetic variants in the cerebrospinal fluid (CSF) were compartmentalized and which variants were shared with the blood plasma. We then measured the viral decay kinetics of individual variants after the initiation of antiretroviral therapy. Compartmentalized HIV-1 variants in the CSF of asymptomatic subjects decayed rapidly after the initiation of antiretroviral therapy, with a mean half-life of 1.57 days. Rapid viral decay was also measured for CSF-compartmentalized variants in four HAD subjects (t_1/2_ mean = 2.27 days). However, slow viral decay was measured for CSF-compartmentalized variants from an additional four subjects with neurological disease (t_1/2_ range = 9.85 days to no initial decay). The slow decay detected for CSF-compartmentalized variants was not associated with poor CNS drug penetration, drug resistant virus in the CSF, or the presence of X4 virus genotypes. We found that the slow decay measured for CSF-compartmentalized variants in subjects with neurological disease was correlated with low peripheral CD4 cell count and reduced CSF pleocytosis. We propose a model in which infiltrating macrophages replace CD4^+^ T cells as the primary source of productive viral replication in the CNS to maintain high viral loads in the CSF in a substantial subset of subjects with HAD.

## Author Summary

Infection of the central nervous system (CNS) with human immunodeficiency virus type 1 (HIV-1) can lead to the development of HIV-1–associated dementia, a severe neurological disease that results in cognitive and motor impairment. Individuals that are chronically infected with HIV-1 sometimes display unique viral variants in their cerebrospinal fluid (CSF) that are not detected in the blood virus population, termed CSF-compartmentalized variants. The cell type that produces CSF-compartmentalized virus throughout the course of infection has not been determined. We used a sensitive assay to detect compartmentalized variants in the CSF of subjects with and without neurological disease, and then measured the decay kinetics of compartmentalized virus when subjects were starting antiretroviral therapy. We found that compartmentalized virus decays rapidly in asymptomatic subjects. Additionally, we detected differential decay (i.e. rapid or slow) in subjects with neurological disease, and this was associated with the number of white blood cells in the CSF. Our data supports a model of HIV-1 infection in the CNS where compartmentalized virus is produced by a long-lived cell type (slow decay), and this virus can be amplified by short-lived cells (rapid decay) that traffic into the CNS, but is increasingly produced from long-lived cells in the immunodeficient state.

## Introduction

Human immunodeficiency virus type 1 (HIV-1)-associated dementia (HAD) is a severe neurological disease that affects a subset of HIV-1-infected individuals [1],[2]. HIV-1 infection of the central nervous system (CNS) occurs shortly after peripheral infection, most likely through the trafficking of infected lymphocytes and monocytes across the blood-brain barrier (BBB) [3],[4]. Once HIV-1 crosses the BBB it can infect perivascular macrophages and brain-resident microglia, and some studies have shown that neurotropic viruses preferentially infect macrophages [5]–[8]. HIV-1 may persist in the CNS during therapy due to the insufficient CNS penetration of some antiretroviral drugs [2], [9]–[11].

HIV-1 variants have been detected at autopsy in the brains of HAD subjects, and these brain-derived variants are genetically distinct from virus detected in the peripheral blood [7], [12]–[15]. A principal impediment to studying viral evolution in the CNS is that direct sampling of HIV-1 in brain tissue is usually possible only once, at biopsy or autopsy. To examine viral populations in the CNS over the course of HIV-1 infection we have relied upon repeated sampling of virus in the cerebrospinal fluid (CSF). Previous studies have shown that virus detected in the CSF originates from both local CNS tissue and the peripheral blood [16]–[19], indicating that the CSF may act as a site of mixing of virus present in the brain and the periphery. In addition, genetic compartmentalization has been reported between blood plasma and CSF viral variants [20]–[23]. We previously examined the cellular sources of HIV-1 in the CNS by utilizing the heteroduplex tracking assay (HTA) to measure viral decay rates in HIV-1-infected subjects initiating antiretroviral therapy [24]. In this study we reported that the subset of compartmentalized virus detected in the CSF of four asymptomatic subjects decayed rapidly after the initiation of therapy, suggesting that the compartmentalized virus is coming from a short-lived cell type, such as CD4^+^ T cells [24].

The population dynamics of systemic HIV-1 replication have been studied extensively [25]–[27], but the extent of viral replication in specific cell types in the CNS over the course of disease is not yet known. The use of antiretroviral drugs to prevent HIV-1 infection of uninfected cells provides a tool for “viewing” the rate of decay for cell-free virus and virally-infected cells. HIV-1 decay in peripheral blood after the initiation of highly active antiretroviral therapy (HAART) occurs in at least two phases [25],[27]. The first phase of decay is rapid and has been proposed to represent the turnover of cell-free virions and productively infected CD4^+^ T cells [25]–[28]. The second phase is slower and may reflect the decay of long-lived infected cells, possibly latently infected resting CD4^+^ T cells and cells of the monocyte lineage [25], [27]–[29], and the release of virions from follicular dendritic cells [28],[30]. Recently, a study using the integrase inhibitor raltegravir reported altered HIV-1 decay kinetics and a reduction of the second viral decay phase [31], suggesting integration as a rate limiting step of infection in a subset of cells. The implications of these data on measured viral decay rates remain to be clarified; however, the reduction in the second phase of HIV-1 decay may indicate that longer-lived HIV-1-infected cells contribute less to total viral load than previously thought, but it does not preclude the possibility that the second phase of HIV-1 decay may reflect the turnover of long-lived cells [31],[32].

In this study, we characterized the lifespan of the cellular source of compartmentalized HIV-1 in the CNS of subjects with and without symptomatic neurological disease by calculating viral decay rates during the initiation of antiretroviral therapy. The heteroduplex tracking assay (HTA) [33],[34] was used to distinguish between HIV-1 genetic variants in the CSF that were either compartmentalized to the CSF or equilibrated with the peripheral blood. HTA has been used in previous studies to differentiate between HIV-1 genetic variants in separate anatomical compartments [22],[24],[35],[36] and HIV-1 evolutionary variants [37]–[42], including drug resistance mutations [43],[44]. The HTA is a useful tool for resolving and quantifying complex viral populations based on their genotype, and is able to detect HIV-1 variants that comprise as little as 1–3% of the total viral population. We targeted the variable regions of the *env* gene for HTA analysis of our subject population in order to resolve multiple HIV-1 genetic variants. In this study we confirm rapid viral decay in the CSF of asymptomatic subjects initiating HAART, and we report reduced rates of viral decay of compartmentalized virus in the CSF in a subset of neurologically symptomatic subjects initiating antiretroviral therapy. These results suggest a shift in the cell type that produces the bulk of the virus in the CSF late in disease as part of the process of viral pathogenesis in the CNS.

## Results

### Subject population characteristics

Our analysis included 11 asymptomatic subjects (7 new subjects, 4 subjects reported in [24]), 1 subject with minor cognitive motor disorder (MCMD), and 7 subjects with HIV-1–associated dementia (HAD; see Table 1). In general, subjects with HAD have higher viral load in the CSF [17],[45],[46] and increased HIV-1 compartmentalization in the CSF [21],[22]. To assess compartmentalization we measured the relative abundance of HIV-1 variants in the blood plasma and CSF as resolved by the heteroduplex tracking assay (HTA), then calculated the percent difference values between the two viral populations (see Table 2). We found that the CSF and plasma viral populations were different for subjects with HIV-associated neurological disease (average = 67% different; range = 36–88% different) compared to the asymptomatic subjects (average = 42% different; range = 10–78% different). This difference approached statistical significance in spite of the small sample size (p = 0.054 using a two-tailed Mann-Whitney test), and this trend of increased viral compartmentalization in the CSF with HAD is consistent with the difference seen in a larger cross-sectional analysis [21]. We next used the HTA to follow differential decay of shared and compartmentalized variants when subjects initiated therapy. In this study, the subjects had an average reduction of 91% of the virus in the blood, and 88% of the virus in the CSF, over the period of sampling for HTA analysis (Table 2).

**Table 1:** Sample characteristics.

Subject ID	Cell counts (cells/µl)		ADC Stage^c^	Drug Regimen	CPE total^d^	HIV-1 RNA (log_10_ copies/ml)^a^	
	CD4^a^	CSF WBC^b^				Plasma	CSF
4012	295	13	0	3TC, NVP, NFV, AZT	2.5	5.18	4.39
4014	1140	20	0	ddI, NVP, d4T	1.5	4.41	4.42
4021	215	2	0	AZT, 3TC, NFV	1.5	4.97	4.02
4022	372	18	0	3TC, NFV, AZT	1.5	4.58	4.77
4023	215	0	0	AZT, 3TC, NFV, EFV	2.0	5.50	3.77
4030	239	5	0	d4T, 3TC, EFV	1.5	4.86	4.06
5005	267	19	0	ABC, NVP, SGC, NFV	2.0	5.30	4.99
4033	173	28	2	IDV, RTV, 3TC, ABC, NVP	3.5	4.83	5.23
4051	344	26	3	AZT, 3TC, EFV	2.0	5.61	5.41
5003	234	39	3	NFV, d4T, ABC	1.5	3.31	4.33
7036	267	150	2	AZT, 3TC, NVP	2.5	5.12	5.37
4013	148	6	1	3TC, NVP, NFV, d4T	2.0	4.62	4.75
4059	53	1	3	AZT, 3TC, LPVr	2.5	5.31	5.08
5002	59	23	3	3TC, NVP, NFV, ABC	2.5	4.24	5.32
7115	50	9	2	AZT, 3TC, LPVr	2.5	5.87	4.85

a Baseline samples.

b Average CSF white blood cell counts over the first 14 days of antiretroviral therapy.

c ADC staging: [68].

d CNS Penetration Effectiveness Rank total. The CPE ranks for each drug in the regimen were summed to get the CPE total.

**Table 2:** HIV-1 variant decay.

Subject ID	% HIV-1 RNA decrease (days on HAART)		Plasma Half-life (days)	CSF-Compartmentalized Variant Data		CSF Shared Variant Data		% Diff.^c^
	Plasma	CSF		% CSF VL^a^	Half-life (days)^b^	% CSF VL^a^	Half-life (days)^b^	
4012	96 (10)	97 (10)	1.36	0	N/A	100	1.73	29
4014	92 (7)	95 (7)	1.96	83.6	1.9	16.4	1.3	37
4021	90 (5)	82 (5)	1.2	7	>1.59^f^	93	1.59	36
4022	87 (4)	93 (4)	1.01	37	0.75	63	0.79	47
4023	90 (3)	79 (3)	0.58	0	N/A	100	0.88	58
4030	90 (3)	90 (3)	1.81	0	N/A	100	1.83	10
5005	97 (10)	98 (10)	2.27	100	2.04	0	N/A	78
4033	92 (8)	92 (8)	1.64	77	1.44	23	2.44	72
4051	95 (10)	94 (10)	2.32	39	2.74	61	2.19	36
5003	54 (6)	82 (6)	2.69^e^	94	1.23	6	1.05	88
7036	99 (25)	99 (25)	2.91	82	3.67	18	2.95	80
4013	93 (15)	20 (9)	2.32	73	28.5	27	4.3	78
		76 (15)			3.9		2.0	
4059	98 (14)	62 (14)	2.35	92	9.85	8	5.84	46
5002	98 (6)	Inc. (6)^d^	1.42	98	No decay	2	No decay	72
		93 (28)			4.24		1.76	
7115	99 (10)	40 (10)	1.25	47	No decay	53	4.88	65
		95 (33)			6.4		6.05	

a Based on the region of *env* (V1/V2 or V4/V5) that was the most reproducible by two independent HTA replicates.

b Reported half-lives are the average calculated from decay analyses of two independent HTA replicates. N/A = not applicable.

c Percent difference values between plasma and CSF viral populations as measured by HTA. Reported values are the average calculated from two independent HTA replicates (see refs. [39],[41] for methods).

d Total CSF viral load increased initially for subject 5002.

e Total plasma viral decay for subject 5003 was calculated for the drop in viral load from days 3 to 6 on HAART. There was a slight increase in plasma viral load from days 6 to 10 on HAART, which can be seen in Figure 2B, but this increase does not seem to be significant. Although the baseline samples were not available for analysis, we know that the baseline plasma viral load was 126,000 copies/ml, and this subject had undetectable viral loads in both plasma and CSF by 2 months post-HAART, so there was an overall good response to antiretroviral therapy. The small variation in plasma viral load from days 6 to 10 on therapy could be explained by a number of technical, pharmacological, and/or biological factors.

f A compartmentalized variant was detected for subject 4021 in the day 5 CSF sample; however, the relative abundance of this variant was less than other bands detected that were not reproducible by HTA, indicating that the detection of this band may be due to inefficient sampling and low viral load. It is equally possible that this band represents a reproducible compartmentalized variant that is decaying more slowly than the other variants detected by HTA. Therefore, the half-life for the CSF-compartmentalized variants is listed as >1.59 days (a half-life of 1.59 days was measured for CSF shared variants).

### Compartmentalized HIV-1 in the CSF of asymptomatic subjects decays rapidly

The HTA is a useful tool for sampling complex viral populations, and is sensitive enough to detect minor variants within the population. We utilized HTAs targeting the hyper-variable regions V1/V2 and V4/V5 of the *env* gene to detect and measure the decay of individual HIV-1 variants in the cerebrospinal fluid and plasma of subjects initiating HAART. The HTA that was the most reproducible (V1/V2 or V4/V5) was used for the final decay and half-life calculations. The half-lives for the different variants in the blood for four of these subjects have been reported previously [47].

**Figure 1:**
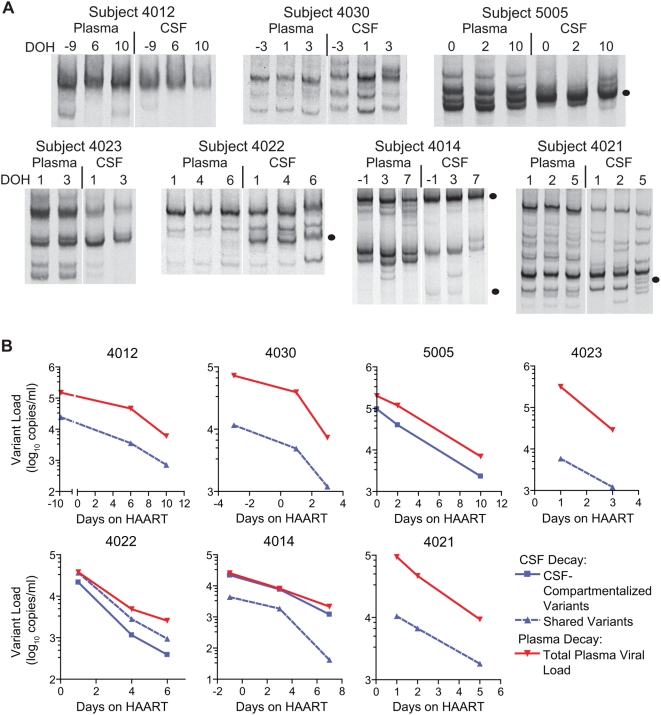
Longitudinal HTA analysis and HIV-1 decay in the blood plasma and CSF of asymptomatic subjects. (A) Longitudinal V1/V2 or V4/V5 HTA analysis of HIV-1 using paired blood plasma and CSF samples from 7 asymptomatic subjects that were initiating antiretroviral therapy. The HTA shown for subject 4014 targeted the V1/V2 region of *env*, and the V4/V5 HTA is shown for all other subjects. Sample time points are listed above each HTA gel as days on HAART (DOH) with day 0 indicating the day that antiretroviral therapy was started. CSF-compartmentalized variants are indicated with a filled black circle next to the gel image. The V4/V5 HTA analysis for subject 4021 revealed a compartmentalized variant in the day 5 CSF sample, not present in the blood plasma, that was reproducible by HTA; however, the relative abundance of this variant (3.6%) was less than that of other bands detected in the same sample that were not reproducible by HTA, indicating that the detection of this band may be due to inefficient sampling and low viral load. Compartmentalized variants were not detected for three subjects. (B) HIV-1 decay kinetics in the blood plasma and CSF. Viral variants in the CSF were categorized as either compartmentalized or shared between the plasma and CSF for the decay analysis. Total plasma viral load decay is shown in red, CSF-compartmentalized variant decay is denoted by the solid blue line, and decay of variants shared between the plasma and CSF is shown by the dashed blue line. It should be noted that in our decay analysis for subject 4012 we assumed the viral load at day 0 would be similar to the viral load measured for the baseline samples at day −9.

The V1/V2 and V4/V5 HTA analyses for the seven new asymptomatic subjects revealed rapid HIV-1 decay for both compartmentalized and shared variants detected in the CSF (see Figure 1). The decay of individual variants was organized into two groups for half-life analysis: decay of CSF-compartmentalized variants and decay of variants shared between the blood and the CSF. The HTA gels for the longitudinal samples from the seven new asymptomatic subjects are shown in Figure 1A, and graphs representing the viral decay are shown in Figure 1B. In this analysis, viral variants that decay more slowly will make up an increasing percentage of the total viral population over the course of therapy. However, if all variants decay at the same rate then the relative percentages will remain the same over time. HIV-1 half-lives for plasma and CSF variants were calculated based on the slopes of the decay curves (summarized in Table 2). Based on data generated from the seven new asymptomatic subjects analyzed in this study, half-lives calculated for the total plasma viral load decay were short (t_1/2_ mean = 1.46 days; t_1/2_ range = 0.58–2.27 days), and total CSF viral load half-lives were short (t_1/2_ mean = 1.5 days; t_1/2_ range = 0.77–2.04 days). These half-lives are similar to the data reported for 4 asymptomatic subjects that were previously studied [24].

Although some asymptomatic subjects have large percent difference values between the blood and CSF viral populations, not all of the variants detected in the CSF met the criteria for compartmentalization. Viral variants in the CSF were considered compartmentalized if they were unique to the CSF or they were present in a substantially higher concentration in the CSF compared to the plasma. CSF-compartmentalized variants were detected in asymptomatic subjects 5005, 4014, and 4022. To increase our sample size we included the half-life data from the four asymptomatic subjects reported in ref. [24] in our analysis of CSF-compartmentalized decay. Including these additional four subjects (n = 7 total asymptomatic subjects with some compartmentalized virus: 3 new subjects and 4 previously reported subjects), we found that the half-lives for CSF-compartmentalized variants in these subjects were short, with a mean of 1.57 days (t_1/2_ range = 0.75–2.75 days; see below). These data indicate that CSF-compartmentalized virus in asymptomatic subjects is most likely originating from a short-lived cell type, such as a CD4^+^ T cell. The reported half-life of a productively infected CD4^+^ T cell is approximately 2 days [28], which coincides with our average measured half-life of 1.57 days in these subjects.

### Differential decay of compartmentalized HIV-1 in the CSF of neurologically symptomatic subjects is correlated with immunodeficiency and CSF pleocytosis

**Figure 2:**
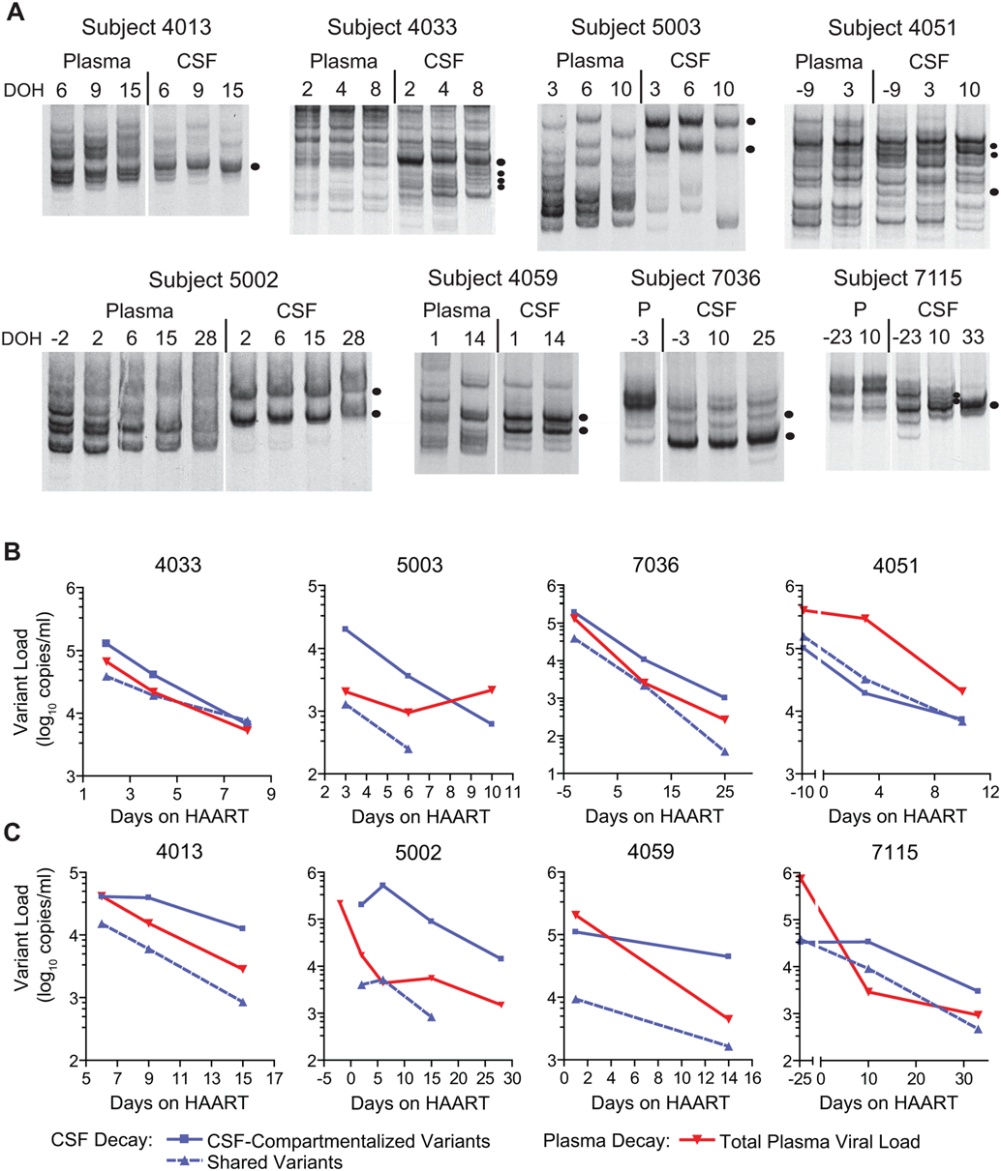
Longitudinal HTA analysis and HIV-1 decay in blood plasma and CSF of neurologically symptomatic subjects. (A) Longitudinal V4/V5 HTA analysis of HIV-1 in paired blood plasma and CSF samples from 1 subject with MCMD (subject 4013) and 7 subjects with HIV-associated dementia that were initiating HAART. Plasma (P) and CSF sample time points are listed above each HTA gel as days on HAART (DOH), and day 0 indicates the start of antiretroviral therapy. CSF-compartmentalized variants are indicated by a filled black circle. (B, C) HIV-1 decay kinetics in the blood plasma and CSF viral populations. Viral variants in the CSF were categorized as either compartmentalized in the CSF or shared between the plasma and CSF. Total plasma viral load decay is shown in red, CSF-compartmentalized variant decay is denoted by the solid blue line, and decay of variants shared between the plasma and CSF is shown by the dashed blue line. Four subjects (4033, 5003, 7036, 4051) displayed rapid decay in their CSF-compartmentalized variant population (shown in panel B), while the other four subjects (4013, 5002, 4059, 7115) had slower decay of CSF-compartmentalized variants (shown in panel C). Subjects 4013, 5002, and 7115 showed differential decay of CSF-compartmentalized variants where initially the compartmentalized variant population decreased very slowly (4013) or increased (5002, 7115) after the start of therapy, and at subsequent time points began decreasing at a faster rate (panel C). In our decay analysis for subjects 4051 and 7115 we assumed that the viral load at day 0 would be similar to the viral load measured for the baseline samples at day −9 for subject 4051 and at day −25 for subject 7115. In this regard, subject 7115 was followed longitudinally for several years prior to the start of antiretroviral therapy, and the viral loads in both the plasma and CSF samples remained relatively constant over time (data not shown).

We expanded our analysis of viral decay to HIV-1-infected subjects who were diagnosed with either MCMD or HAD to address the hypothesis that CSF-compartmentalized variants in these subjects originate from longer-lived cells. Viral decay in the CSF of eight subjects with neurological disease was analyzed using HTAs targeting the V1/V2 and V4/V5 regions of *env*. The HTA analyses for the eight subjects with HIV-associated neurological disease showed either rapid or slow viral decay among the subjects. The longitudinal HTA gels for each neurologically symptomatic subject are shown in Figure 2A, and the graphs of viral decay are shown in Figure 2B and 2C. Similar to the asymptomatic subject decay analysis, individual variants were grouped as either CSF-compartmentalized variants or variants shared between the blood and the CSF for the decay analysis.

**Figure 3:**
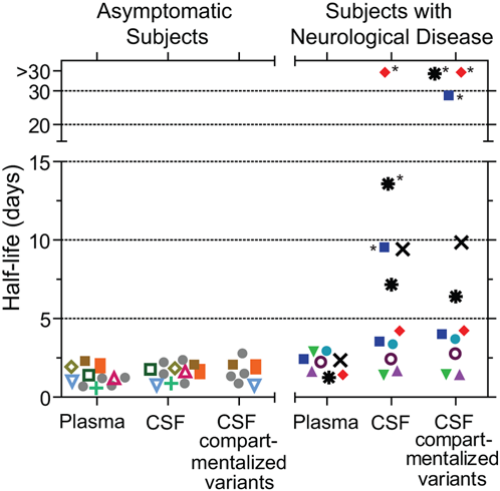
Summary of HIV-1 half-lives from asymptomatic and neurologically symptomatic subjects. Half-lives were calculated for total plasma viral load decay (Plasma), total CSF viral load decay (CSF), and CSF-compartmentalized variant decay (CSF compartmentalized variants). The half-lives were calculated using the time points when the variant load initially dropped after each subject initiated antiretroviral therapy. All half-lives listed in the figure are the average calculated from decay analyses of two independent HTA replicates. Half-lives are listed for the seven new asymptomatic subjects analyzed in this study plus four asymptomatic subjects (labeled as 4 Asy.) that were previously reported [24], 1 subject with MCMD (subject 4013), and 7 subjects with HIV-1–associated dementia. The asymptomatic subjects include: 4012 (open green square), 4030 (open yellow-green diamond), 5005 (brown square), 4023 (green plus symbol), 4022 (open, inverted blue triangle), 4014 (orange rectangle), 4021 (open fuchsia triangle), and 4 Asy. (gray circles). The subjects with HIV-associated neurological disease include: 4033 (purple triangle), 5003 (inverted green triangle), 7036 (teal circle), 4051 (open purple circle), 4013 (blue square), 5002 (red diamond), 4059 (black×symbol), and 7115 (black irregular circle). Two different half-lives are listed for subjects 4013, 5002, and 7115 for both total CSF viral decay (CSF) and CSF-compartmentalized variant decay (CSF-compartmentalized variants). Each of these three subjects showed biphasic decay by HTA analysis, and the half-life data point denoted with the asterisk represents the phase 1 half-life (slower), while the half-life lacking the asterisk was calculated for phase 2 of decay (faster). The CSF-compartmentalized variant population increased initially for subjects 5002 and 7115, so there was no decay detected for these subjects during phase 1 and their half-lives are listed on the graph as >30 days. Similarly, the total CSF viral load increased for subject 5002 during phase 1 of decay and the half-life is listed on the graph as >30 days.

Total plasma viral load decay was rapid for all subjects with neurological disease, with a mean half-life of 2.11 days (t_1/2_ range = 1.42–2.91 days; summarized in Table 2 and Figure 3). We measured rapid viral decay for CSF-compartmentalized variants after the initiation of HAART for four subjects with HAD (4033, 5003, 7036, 4051; t_1/2_ mean = 2.27 days; t_1/2_ range = 1.23–3.67 days; Figure 2B and Figure 3; summarized in Table 2), similar to asymptomatic subjects. In contrast, prolonged viral decay was measured for CSF-compartmentalized variants for the other four subjects with neurological disease (4013, 5002, 4059, 7115; t_1/2_ range = 9.85 days to no initial decay), with three subjects displaying biphasic decay. CSF-compartmentalized variants for subjects 4013, 5002, and 7115 displayed a biphasic decay (see Figure 2C), where the first phase of viral decay was slow (4013 t_1/2_ = 28.5 days; 7115 t_1/2_ = no initial decay; 5002 t_1/2_ = no initial decay), and the second phase was faster (4013 t_1/2_ = 3.9 days; 7115 t_1/2_ = 6.4 days; 5002 t_1/2_ = 4.24 days). Figure 3 and Table 2 report the half-lives calculated for both phases of decay. Subject 4059 displayed only a slower decay rate for the CSF-compartmentalized variants (t_1/2_ = 9.85 days). Total CSF viral load decay was similar to the decay rates measured for CSF-compartmentalized variants for all subjects with neurological disease. This is due to the fact that most of the virus in the CSF was compartmentalized in these HAD subjects. The decay of the small amounts of shared variants fluctuated in these subjects from decreasing with a rate similar to the virus in plasma to decreasing with a slow rate similar to that of the CSF-compartmentalized variants (see Table 2).

For each CSF sample time point the CSF white blood cell (WBC) count was measured to determine if any subjects had CSF pleocytosis (defined as >5 cells/µl; [48],[49]). We found that all four subjects with rapid CSF-compartmentalized variant decay either had high CSF WBC levels at entry (4033 = 28 cells/µl; 5003 = 46 cells/µl; 7036 = 240 cells/µl; 4051 = 12 cells/µl), or the CSF WBC levels increased while on therapy. Conversely, the four subjects with neurological disease that displayed slower CSF-compartmentalized variant decay either had extremely low levels of CSF WBCs at entry (4013 = 10 cells/µl; 5002 = 66 cells/µl; 4059 = 1 cells/µl; 7115 = 12 cells/µl), or the CSF WBC levels decreased to low levels after the initiation of antiretroviral therapy. We examined the CSF WBC levels of these two groups in more detail by calculating the CSF WBC average for each subject from baseline through the first 14 days of antiretroviral therapy. The subjects with rapid CSF-compartmentalized variant decay had higher CSF WBC averages, while subjects with slower or biphasic CSF-compartmentalized variant decay had lower CSF WBC averages (see Table 1), and this difference was statistically significant (p = 0.029 using a two-tailed Mann-Whitney test). It has been reported that HIV-1-infected subjects with CD4 counts below 50 cells/µl have reduced CSF pleocytosis [49]. We also examined whether the viral decay rates measured by HTA were correlated with the degree of immunodeficiency by analyzing CD4 counts for each group of subjects. The four subjects with rapid CSF-compartmentalized variant decay had significantly higher baseline CD4 counts (see Table 1) compared to the four subjects with slower CSF-compartmentalized variant decay (p = 0.006 using a two-tailed unpaired t-test). Thus, in subjects with HIV-1–associated neurological disease, viral decay rates are associated with the degree of immunodeficiency and CSF pleocytosis.

We did not detect an association between CSF pleocytosis and rapid viral decay in the CSF for asymptomatic subjects. The CSF WBC average was calculated for each subject as stated above, and the range extended from 0 cells/µl up to 20 cells/µl (Table 1). All variants detected in the CSF of asymptomatic subjects decayed rapidly upon the initiation of antiretroviral therapy; however, we found that the presence of CSF-compartmentalized variants was associated with higher average CSF WBC levels. All four of the asymptomatic subjects that did not have compartmentalized virus had low average CSF WBC counts (4012, 4030, 4023, 4021), while the three asymptomatic subjects that had detectable CSF-compartmentalized variants also had higher average CSF WBC levels (5005, 4022, 4014; see Table 1). Thus in the asymptomatic subjects the presence of pleocytosis may be associated with an early inflammatory response to increased levels of autonomously replicating virus.

### Slower decay in neurologically symptomatic subjects is not associated with CNS drug penetration, drug resistance mutations, or V3/X4 sequence differences

Some antiretroviral drugs have poor penetration into the CNS [50]. In order to determine whether the differential decay we detected by HTA was associated with poor CNS drug penetration, we calculated the CNS Penetration Effectiveness (CPE) rank [50] for the drug regimens that each of the 15 subjects were receiving at the time of sample collection (see Table 1). Drugs that have poor penetration into the CNS were assigned a rank of 0, intermediate penetration was assigned a rank of 0.5, and high penetration was assigned a rank of 1 [50]. The four subjects that showed a longer viral half-life by HTA analysis had CPE ranks ranging from 2.0 to 2.5, while the other subjects that displayed rapid viral decay had CPE ranks from 1.5 (5 subjects) to 3.5 (1 subject). All subjects with neurological disease had CPE ranks above 2.0 except for subject 5003 (CPE rank = 1.5). A previous study reported that CPE ranks below 2.0 were associated with a significant (88%) increase in the ability to detect virus in the CSF, and higher CSF viral loads were associated with low CPE ranks [50]. All of the subjects with longer viral half-lives had CPE ranks of 2.0 or above, suggesting that the slower HIV-1 decay we detected by HTA was not associated with poor CNS drug penetration. Alternatively, there could be infected cells located in parenchymal compartments that are less accessible to drugs, but this seems unlikely because the virus still has access to the CSF.

We also investigated the possibility that slower decay was a result of drug resistance mutations present in the viral population in the CSF. Drug resistance mutations were measured for CSF samples of subjects 4013, 5002, 4059, and 7115. The resistance test was conducted for time points after the initiation of drug selection to allow for enrichment of any potential drug resistant variants. Subjects 4013, 5002, and 4059 showed no evidence of resistance mutations in reverse transcriptase (RT) or protease that confers resistance to antiretroviral drugs (data not shown). Subject 7115 had the resistance mutation K103N in RT, which confers resistance to non-nucleoside RT inhibitors (NNRTI). However, at the time of this study, subject 7115 was not taking an NNRTI, and was instead on a drug regimen that included zidovudine, lamivudine, and lopinavir. Therefore, there is no evidence that drug resistance played a role in the slower viral decay detected by HTA in these four subjects.

**Figure 4:**
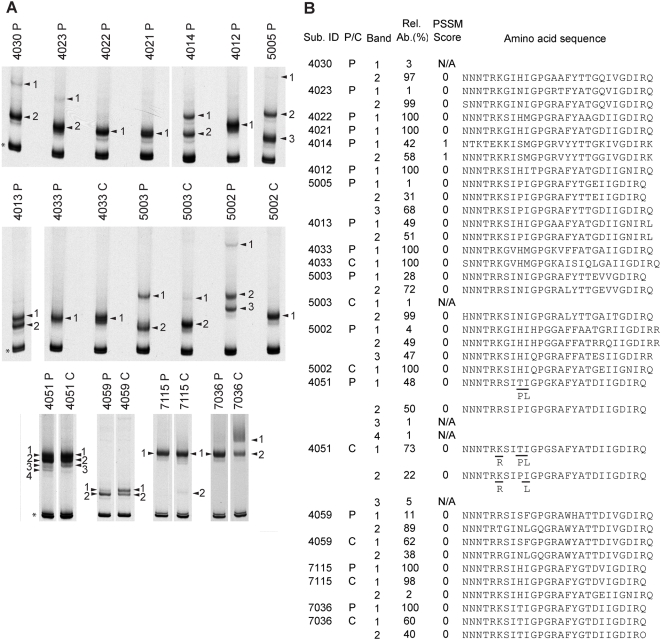
Examination of the V3 region of *env* using the biotin-V3 HTA. (A) Biotin-V3 HTA analysis of the baseline samples collected for asymptomatic and neurologically symptomatic subjects. Plasma samples (P) were analyzed for all subjects, and CSF samples (C) were analyzed for HAD subjects. The asterisk indicates the double-stranded probe band, and shifted heteroduplex bands are noted with arrowheads and numbers. (B) Summary table of the V3 sequence information obtained using the biotin-V3 HTA procedure. The desired V3 bands were excised from the dried gel, the query strand DNA was purified and PCR amplified, and the V3 PCR products from each band were sequenced. The sample type (P/C; P = plasma sample, C = CSF sample), relative abundance (Rel. Ab.), and PSSM score (0 = R5-like sequence, 1 = X4-like sequence, N/A = not applicable) for each sequence are listed in the table. Note: the V3 PCR primers used to amplify the V3 products extend into the first four and the last three amino acids of the V3 sequence. The V3 sequences provided do not include these amino acids; however, we added the JRFL consensus amino acid sequence to the beginning (CTRP) and end (AHC) of each V3 sequence to predict coreceptor usage using PSSM. Mixtures in the sequence peaks were detected at specific positions in V3 bands from subject 4051 plasma and CSF samples, and these mixtures are noted in the reported amino acid sequences.

Using the biotin-V3 HTA procedure, we also examined whether slower viral decay was associated with V3 sequence differences. The biotin-HTA is a modification of the original HTA method that incorporates a biotin tag into the probe to allow direct sequencing of the query strand isolated from the gel [51]. This newly developed HTA procedure resolves minor variants in the gel, and then allows the recovery and sequence analysis of both major and minor HIV-1 V3 variants from complex viral populations [51]. Following V3 PCR amplification and HTA analysis, we excised the gel fragments containing the V3 heteroduplexes, purified the query DNA strand using streptavidin-coated magnetic Dynabeads®, and directly sequenced the subsequent V3 PCR products [51]. The migration patterns for the V3 heteroduplexes and the inferred V3 amino acid sequence obtained for the heteroduplex in each gel band are shown in Figure 4. The biotin-V3 HTA procedure was conducted on plasma samples from all subjects at the first time point collected, and CSF samples were analyzed for subjects with HIV-associated dementia. No significant V3 sequence differences were detected between asymptomatic and symptomatic subjects, or between subjects with rapid versus slow decay by HTA (Figure 4B). Only one subject (4014) had V3 sequences that were X4-like by the Position-Specific Scoring Matrix (PSSM) method [52] of predicting co-receptor usage based on genotype. We did note that two subjects with slower decay by HTA had compartmentalized V3 variants detected in the CSF viral population that were much more R5-like by sequence compared to the V3 sequence variants detected in the plasma viral population. However, R5-like V3 sequences were also detected in the CSF for HAD subjects with rapid viral decay, indicating that V3 sequence differences and co-receptor usage are not responsible for the differential decay detected by HTA.

## Discussion

There are several lines of evidence that support the idea that HIV-1 can replicate in the central nervous system (CNS). HIV-1-infected macrophages and microglia have been detected in the brains of subjects with HIV-1–associated dementia (HAD) at autopsy [6],[53],[54]. In addition, genetically distinct HIV-1 variants, different from those in the peripheral blood, are seen in the CNS of subjects with HAD [7], [12]–[15]. These inferences can be extended using CSF as a surrogate for the CNS where genetic compartmentalization can be detected when comparing blood and CSF viral variants [20]–[23], and bulk virus in the CSF of subjects initiating HAART can decay with different kinetics compared to virus in the blood [16],[19],[55]. Furthermore, it appears that this independent replication is relevant, if not causal, of HIV-associated neuropathogenesis. The extent of compartmentalization in the CSF, as measured by the heteroduplex tracking assay, increases in subjects with HAD, suggesting more sustained autonomous replication is associated with the neurological disease state [21],[22]. Also, slow decay of virus in the CSF compared to the blood is associated with subjects with neurological disease, especially HAD subjects, suggestive of virus being produced from a different cellular source [16],[19],[55]. In addition to viral genetic compartmentalization there are other markers of neuropathogenesis in HIV-1-infected individuals, such as CSF neopterin [56],[57], CSF light-chain neurofilament protein [57]–[59], and CSF chemokine levels [60]–[64]. In the current work we have attempted to combine the observations of viral genetic compartmentalization and differential decay in subjects initiating HAART by comparing the rates of decay of variants shared between the CSF and the blood versus those variants that were compartmentalized in the CSF. The goal of this work was to examine the link between compartmentalized virus as a marker for autonomous replication in the CNS and the production of virus in the CNS by long-lived cells.

We used heteroduplex tracking assays (HTAs) targeting the variable regions of *env* to identify CSF-compartmentalized variants and variants shared between the CSF and blood plasma, and then measured the viral decay kinetics of these two distinct classes of viral variants after the initiation of antiretroviral therapy for asymptomatic and neurologically symptomatic subjects. We found that plasma HIV-1 variants decayed rapidly for both neurologically asymptomatic and symptomatic subjects, indicating that short-lived cells, presumably activated CD4^+^ T cells, are the predominant source of virus in the periphery during all disease stages. Additionally, shared and compartmentalized variants in the CSF of seven asymptomatic subjects decayed rapidly, with a mean half-life of 1.35 and 1.57 days, respectively. These decay rates are consistent with our previous study of four asymptomatic subjects [24]. HIV-1 viral load decays in the peripheral blood with the same half-life as a productively infected CD4^+^ T cell (approximately 2 days; [25],[27],[28]), so it is most likely that CSF-shared and compartmentalized virus in asymptomatic subjects is originating from a short-lived cell type, such as a CD4^+^ T cell. The level of HIV-1 compartmentalization in the CSF in these asymptomatic subjects varied, and we noted that there was a trend of increased CSF pleocytosis in the asymptomatic subjects with greater compartmentalization.

We also examined HIV-1 decay in subjects with neurological disease that were starting HAART. Rapid viral decay was measured for CSF-compartmentalized variants after the initiation of HAART for four HAD subjects (t_1/2_ mean = 2.27 days), while slow viral decay was measured for CSF-compartmentalized variants from the other four subjects with neurological disease (t_1/2_ range = 9.85 days to no initial decay). It is known that HIV-1 may persist in the CNS during antiretroviral therapy due to insufficient CNS penetration of some antiretroviral drugs [2], [9]–[11]. We determined that the slow decay detected for CSF-compartmentalized variants was not associated with poor CNS drug penetration, the presence of drug resistant virus in the CSF, or the detection of X4-like virus genotypes. It has been suggested that HIV-1 produced by long-lived cell lineages such as macrophages, microglia, and resting CD4^+^ T cells most likely decays with a half-life of 14 days or greater [27]–[29]. The longer half-lives we detected suggest that compartmentalized HIV-1 in the CSF of some neurologically symptomatic subjects may be originating from a long-lived cell type.

While slower HIV-1 decay was detected for half of the subjects with neurological disease, compartmentalized variants in the CSF of some subjects decayed rapidly. Further analysis revealed that the differential decay measured for CSF-compartmentalized variants in subjects with neurological disease was correlated with the degree of CSF pleocytosis. Four of the eight subjects with HIV-associated neurological disease displayed rapid CSF-compartmentalized variant decay, and this was correlated with higher CSF WBC levels (moderate to severe pleocytosis). The compartmentalized variants detected in the CSF of the four other subjects showed slow or biphasic decay after the initiation of HAART, and this was associated with lower CSF WBC levels (no or mild pleocytosis). Additionally, the subjects with rapid CSF-compartmentalized variant decay had significantly higher CD4 counts than subjects with slow compartmentalized variant decay, indicating that subjects with slow decay of CSF-compartmentalized virus have increased immunodeficiency. We suggest that more profound immunodeficiency results in fewer lymphocytes trafficking into the CNS, which is consistent with the decreased CSF WBC counts for the subjects with slow decay. HIV-1 infection can be associated with CSF pleocytosis in neurologically symptomatic subjects, asymptomatic subjects, and individuals lacking any CNS opportunistic infections [48]. Additionally, some studies have shown that CSF WBC levels are correlated with CSF HIV-1 RNA concentrations [49],[65],[66], and CSF pleocytosis has been shown to decrease after the initiation of antiretroviral therapy [48]. In this current study we found an association between the extent of immunodeficiency, CSF pleocytosis and rapid HIV-1 decay kinetics for compartmentalized variants in the CSF of neurologically symptomatic subjects, although the strength of the interpretation is somewhat limited by our small sample size.

**Figure 5:**
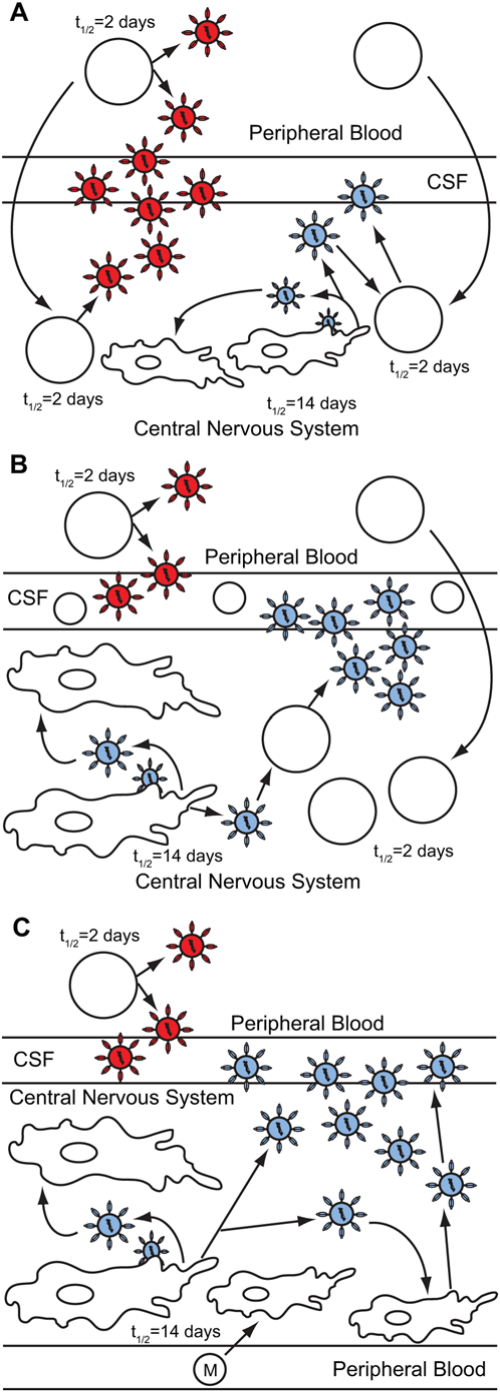
Model of HIV-1 infection in the central nervous system. CD4^+^ T cells are represented by open circles, macrophages are represented by the irregularly shaped cells, monocytes are represented by open circles labeled with an M, blood plasma viral variants are represented by the red virus particles, and CNS compartmentalized viral variants are represented by the blue virus particles. (A) HIV-1 infection in the CNS of a subset of asymptomatic subjects without detectable compartmentalized virus or CSF pleocytosis. All HIV-1 detected in the CSF decays rapidly after the initiation of therapy, suggesting that CSF virus is coming from a short-lived cell type. (B) HIV-1 infection in the CNS of asymptomatic and neurologically symptomatic subjects with compartmentalization, high CSF pleocytosis and rapid viral decay. In this model, local CNS virus is able to replicate to higher titers during periods of immunodeficiency and stimulate an inflammatory response in the CNS. Uninfected CD4^+^ T cells that migrate into the CNS can become infected by compartmentalized HIV-1 produced by macrophages and microglia in the CNS, and then amplify the local CNS-compartmentalized virus to higher concentrations. Thus, a rapid decay rate for compartmentalized virus is detected after the initiation of antiretroviral therapy. (C) HIV-1 infection in the CNS of neurologically symptomatic subjects with slow viral decay. Our data indicate that compartmentalized variants in the CSF of HAD subjects are originating from long-lived macrophages and microglia in the CNS, resulting in a slow decay rate for compartmentalized virus. We propose that periods of profound immunodeficiency allow compartmentalized virus in the CNS to replicate to high titers, and that in the absence of other lymphocytes (such as CD4^+^ T cells) peripheral, uninfected monocytes may migrate into the brain parenchyma in large numbers and differentiate into perivascular macrophages. These macrophages can then become infected by compartmentalized HIV-1 variants in the CNS and support viral replication at detectable levels.

Taken together, we have developed a model of HIV-1 infection in the CNS in the context of neurological disease (Figure 5). The model has several features that incorporate viral genetic compartmentalization, CSF pleocytosis, and viral decay rates in the CSF as a measure of the virus-producing cell. First, the majority of the virus detected in the CSF of a subset of asymptomatic subjects is imported from the peripheral blood (Figure 5A). HIV-1-infected CD4^+^ T cells in the peripheral blood release virus that is detectable in the blood plasma and the CSF and that decays rapidly upon the start of antiretroviral therapy, representing the relatively fast turnover of uninfected CD4^+^ T cells. HIV-1-infected CD4^+^ T cells in the peripheral blood can migrate from the periphery into the CNS and secrete virus in the CNS that is genetically similar to virus in the peripheral blood. No or only mild pleocytosis was detected for this group of asymptomatic subjects, and we suggest this represents minimal inflammation in the CNS. It is possible that some CNS HIV-1 variants are independently replicating at a low level in these asymptomatic subjects, but we were not able to detect these genetic variants above the background of virus recently imported from the periphery. In these subjects virus decays with the half life of peripheral T cells, the presumed source of the virus.

A second pattern exists for the other asymptomatic subjects and also for a subset of the neurologically symptomatic subjects. There is increased compartmentalization of HIV-1 in this subset of asymptomatic subjects, and the majority of virus detected in the CSF is compartmentalized in HIV-1-infected individuals with severe neurological disease. In addition, both of these groups have increased pleocytosis. We found that CSF-compartmentalized variants decayed rapidly upon the initiation of antiretroviral therapy in these remaining asymptomatic subjects and in this subset of four subjects with HIV-1–associated dementia. It is possible that compartmentalized variants detected in these subjects are produced by long-lived cells in the CNS; however the majority of the compartmentalized virus is produced by a short-lived cell type. We propose that compartmentalized virus may be maintained by long-lived cells in the CNS and that this virus is amplified by short-lived trafficking CD4^+^ T cells to detectable levels in the CSF for asymptomatic subjects, and to high titers in the CSF of HAD subjects (Figure 5B). The elevated level of pleocytosis is indicative of an inflammatory response, most likely to the autonomously replicating virus. Increased levels of CSF white blood cells may account for the influx of T cells that could be the source of the short-lived cells that are amplifying the compartmentalized virus. We would expect that most of the infiltrating T cells are HIV-specific, although some lymphocytes may be migrating into the CNS due to a general inflammatory environment. The asymptomatic subjects in this group have the hallmarks of viral pathogenesis associated with neurological disease and may be at risk for transition to HAD.

Third, we detected slow decay of compartmentalized variants in the CSF for the four remaining subjects with neurological disease. These subjects shared the feature of viral genetic compartmentalization but did not show high levels of pleocytosis. Additionally, this subject group had the lowest blood CD4^+^ T cell counts (Table 1), indicating a state of increased immunodeficiency. We suggest that these subjects have more profound immunodeficiency, which would allow even more extensive viral replication and compartmentalization in the CNS (Figure 5C). Increased immunodeficiency would result in reduced trafficking of CD4^+^ T cells into the CNS, so these cells would no longer be present to amplify virus from local CNS tissue, consistent with the reduced pleocytosis in this group. The slow decay rate of virus in the CSF in the absence of inflammatory cells suggests that compartmentalized HIV-1 in the CNS of these HAD subjects is originating from a long-lived cell type, such as perivascular macrophages and/or microglia in the CNS. Virus is unlikely to be coming from T cells that are persisting in the absence of immune-mediated killing since there is still rapid viral decay in the peripheral blood. The CSF viral loads of all four subjects displaying slow decay were high, similar to subjects with rapid viral decay, suggesting that a large amount of compartmentalized virus is being produced by longer-lived cells in the CNS. This may suggest that peripheral, uninfected monocytes may migrate into the brain parenchyma and differentiate into perivascular macrophages to levels that can sustain high viral loads in the CSF. An influx of monocytes into the CNS could also allow the entrance of peripherally-infected monocytes, which would explain the slower decay we detected for shared variants in the CSF of these subjects.

Our studies support a model where increasing levels of autonomous viral replication in the CNS first induces an inflammatory state that then progresses to neurologic disease with increasing immunodeficiency. More profound immunodeficiency ultimately reveals long-lived cells that are able to maintain independent replication of virus in the CNS. Several *env* gene markers have been described in viral sequences taken at autopsy and linked to the ability of HIV-1 to infect macrophages [12],[13]. The CSF provides an alternative window on these viral sequences where the evolution of the virus and its properties can be followed over time and into the disease state. Viral genetic compartmentalization and other markers of CNS inflammation could also play an important role in defining subjects at risk of progression to neuropathogenesis in the absence of therapeutic intervention.

## Materials and Methods

### Ethics statement

This study was conducted according to the principles expressed in the Declaration of Helsinki. The study was approved by the Institutional Review Board of the University of California at San Francisco. All subjects provided written informed consent for the collection of samples and subsequent analysis.

### Subject population and sampling

The samples from study subjects used for variant decay analysis were collected during previous studies carried out at the University of California at San Francisco. All subjects used in this study were HIV-1-infected subjects that were initiating highly-active antiretroviral therapy. Subjects 4012, 4013, 4014, 5002, 5003, and 5005 were recruited from a study examining antiretroviral therapy responses in the CSF, and are described in more detail in ref. [67]. Serial blood plasma and cerebrospinal fluid (CSF) samples were collected at baseline prior to the start of therapy and at varying intervals thereafter. Plasma and CSF HIV-1 RNA concentrations were determined using the Amplicor HIV Monitor kit (Roche). CSF white blood cell counts were measured by routine methods in the San Francisco General Clinical Laboratory. Drug resistance mutations were analyzed for CSF samples of subjects 4013, 5002, 4059, and 7115 using the TRUGENE® HIV-1 Genotyping Test Resistance Report using GuideLines™ Rules 12.0 (Bayer HealthCare).

### RNA isolation, RT–PCR, and HTA

Viral RNA isolation, RT–PCR, and HTA procedures were conducted as previously described [24], [39]–[41]. Briefly, viral RNA was isolated from blood plasma and CSF samples (140 µl) using the QIAmp Viral RNA kit (Qiagen). Prior to RNA isolation, all CSF samples were centrifuged at 2,500 rpm for 5 minutes to remove any contaminating cellular debris. Samples with viral RNA levels less than 10,000 copies/ml were pelleted (0.5–1.0 ml) by centrifugation at 25,000×g for 1.5 hours prior to RNA isolation to increase template number and improve sampling. Reverse transcription and PCR amplification of the V1/V2, V3, and V4/V5 regions of *env* were conducted with 5 µl of purified RNA (from 60 µl column elution volume) using primers that have been previously described for V1/V2 [39],[41], V3 [51]; and V4/V5 [41] and using the Qiagen One-Step RT-PCR kit (Qiagen) as per manufacturer's instructions.

Heteroduplex annealing reactions were conducted as previously described [39],[40]. The heteroduplexes were separated by 6% native polyacrylamide gel electrophoresis for V1/V2 and V4/V5 HTA [24],[39], and by 12% PAGE for biotin-V3 HTA [51]. The HTA probes used in these studies have been previously reported: V1/V2 Ba-L probe [39],[41], V1/V2 JRFL probe [39],[41], V4/V5 NL4-3 probe [24], V4/V5 YU2 probe [41], and the V3 Mut-1 probe [51]. The HTA gels were dried under vacuum, and bands were visualized by autoradiography. For the biotin-V3 HTA procedure, the desired labeled bands were excised from the dried gels, the DNA was purified from the gel, and the V3 sequence was obtained as previously described [51]. Duplicate RT-PCR products were analyzed by HTA for each sample to validate sampling and ensure reproducibility of the HTA pattern at each time point. Any time points where the HTA pattern between the two replicates differed significantly (>20%) were not used in the data analysis. Percent difference values between plasma and CSF viral populations were calculated as previously described [39],[41].

### Phosphorimager analysis and half-life calculations

The dried HTA gels were exposed to a PhosphorImager screen, and the relative abundance of each detected viral variant (heteroduplex) was calculated using ImageQuant software (Molecular Dynamics). The variant RNA concentration was calculated by multiplying the relative abundance of each individual variant by the total HIV-1 RNA concentration for that sample. Variants in the CSF were considered compartmentalized by HTA if they were either unique to the CSF or if they had a substantially higher copy number in the CSF compared to the plasma. Compartmentalized variant half-lives were calculated using the time points when the viral load initially dropped after the start of antiretroviral therapy.
